# Examining the Timeliness of ST-elevation Myocardial Infarction Transfers

**DOI:** 10.5811/westjem.2020.8.47770

**Published:** 2021-02-15

**Authors:** Michael J. Ward, Timothy J. Vogus, Daniel Muñoz, Sean P. Collins, Kelly Moser, Cathy A. Jenkins, Dandan Liu, Sunil Kripalani

**Affiliations:** * Vanderbilt University Medical Center, Department of Emergency Medicine, Nashville, Tennessee; † VA Tennessee Valley Healthcare System, Department of Emergency Medicine, Murfreesboro, Tennessee; ‡ Vanderbilt University, Owen Graduate School of Management, Nashville, Tennessee; § Vanderbilt University School of Medicine, Division of Cardiology, Nashville, Tennessee; ¶ Vanderbilt University School of Medicine, Department of Biostatistics, Nashville, Tennessee; || Vanderbilt University Medical Center, Section of Hospital Medicine, Division of General Internal Medicine and Public Health, Department of Medicine, Center for Clinical Quality and Implementation Research, Nashville, Tennessee

## Abstract

**Introduction:**

Despite large-scale quality improvement initiatives, substantial proportions of patients with ST-elevation myocardial infarction (STEMI) transferred to percutaneous coronary intervention centers do not receive percutaneous coronary intervention within the recommended 120 minutes. We sought to examine the contributory role of emergency medical services (EMS) activation relative to percutaneous coronary intervention center activation in the timeliness of care for patients transferred with STEMI.

**Methods:**

We conducted a retrospective analysis of interfacility transfers from emergency departments (ED) to a single percutaneous coronary intervention center between 2011–2014. We included emergency department (ED) patients transferred to the percutaneous coronary intervention center and excluded scene transfers and those given fibrinolytics. We calculated descriptive statistics and used multivariable linear regression to model the association of variables with ED time intervals (arrival to electrocardiogram [ECG], ECG-to-EMS activation, and ECG-to-STEMI alert) adjusting for patient age, gender, mode of arrival, weekday hour presentation, facility transfers in the past year, and transferring facility distance.

**Results:**

We identified 159 patients who met inclusion criteria. Subjects were a mean of 59 years old (standard deviation 13), 22% female, and 93% White; 59% arrived by private vehicle, and 24% presented after weekday hours. EDs transferred a median of 9 STEMIs (interquartile range [IQR] 3, 15) in the past year and a median of 65 miles (IQR 35, 90) from the percutaneous coronary intervention center. Median ED length of stay was 65 minutes (IQR 51, 85). Among component intervals, arrival to ECG was 6%, ECG-to-EMS activation 32%, and ECG-to-STEMI alert was 49% of overall ED length of stay. Only 18% of transfers had EMS activation earlier than STEMI alert. ECG-to-EMS activation was shorter in EDs achieving length of stay ≤60 minutes compared to those >60 minutes (12 vs 31 minutes, P<0.001). Multivariable modeling showed that after-hours presentation was associated with longer ECG-to-EMS activation (adjusted relative risk [RR] 1.05, P<0.001). Female gender (adjusted RR 0.81, P<0.001), prior facility transfers (adjusted RR 0.84, P<0.001), and initial ambulance presentation (adjusted RR 0.93, P = 0.02) were associated with shorter ECG-to-EMS activation.

**Conclusion:**

In STEMI transfers, faster EMS activation was more likely to achieve a shorter ED length of stay than a rapid, percutaneous coronary intervention center STEMI alert. Large-scale quality improvement efforts such as the American Heart Association’s Mission Lifeline that were designed to regionalize STEMI have improved the timeliness of reperfusion, but major gaps, particularly in interfacility transfers, remain. While the transferring EDs are recognized as the primary source of delay during interfacility STEMI transfers, the contributions to delays at transferring EDs remain poorly understood.

## INTRODUCTION

There is evidence that early initiation of emergency medical services (EMS) prior to activation of the cardiac catheterization laboratory at the percutaneous coronary intervention center may reduce the time spent at transferring emergency departments (ED).^4^ However, the role that EMS plays in transfer timeliness compared with other ST-elevation myocardial infarction (STEMI) transfer processes is unclear. With EMS agencies and percutaneous coronary intervention centers requiring separate activation during a STEMI, the transferring ED must choose which process step to perform first. Thus, we sought to evaluate this decision and how the timing of transferring ED activation of EMS when compared with percutaneous coronary intervention center activation influenced the timeliness of interfacility transfers for patients with suspected STEMI.

## METHODS

### Study Design and Population

Vanderbilt University Medical Center (VUMC) is a quaternary care center for cardiovascular services in Middle Tennessee that provides 24/7 primary percutaneous coronary intervention capabilities and medical and surgical management of cardiovascular conditions. VUMC has a catchment area over 65,000 square miles and receives interfacility transfers from dozens of referring EDs in the region. Primary percutaneous coronary intervention activation at VUMC activates the cardiac catheterization team and prepares the laboratory for intervention. After hours, staff must be available onsite within 30 minutes. For the transferring ED, EMS must be activated separately and no formal policy exists regarding the order of such decisions.

In this study we sought to examine the contributory role of each activity to overall STEMI transfer timeliness. We included patients with suspected STEMI who experienced interfacility transfer from an outside ED to VUMC between January 1, 2011–December 31, 2014. We excluded the following patients: 1) those who received fibrinolytics, which are recommended when the patient presents to a non-percutaneous coronary intervention facility and the anticipated delay to primary percutaneous coronary intervention is ≥120 minutes (class I, level of evidence A)^8^; 2) those who were transported directly to VUMC from the field; 3) were initially transferred for reasons other than STEMI; 4) did not receive a cardiac catheterization; and 5) were missing transfer ED health records or had incomplete transferring ED operational data (eg, arrival timestamp). This study was approved by the VUMC Institutional Review Board.

### Data Collection

We developed a data dictionary and performed dual abstractor data collection using REDCap, a secure, browser-based, metadata-driven electronic data capture tool.^9^ Data were abstracted from health records from transferring EDs that are regularly collected and stored in VUMC’s electronic health record. Operational data included the following: transferring hospital; transferring ED timestamps (arrival, diagnostic electrocardiogram [ECG]; physician evaluation; percutaneous coronary intervention center activation; EMS activation; EMS arrival, exit); percutaneous coronary intervention center arrival; and percutaneous coronary intervention start (ie, initiation of cardiac catheterization). We also classified facilities as rural/urban using the Rural Urban Commuting Area codes,^10^ presence in the middle Tennessee regional STEMI network, and driving distance to VUMC (using Google Maps). Clinical data included presenting symptoms, demographics, comorbidities, and 30-day mortality.

**Population Health Research Capsule**

What do we already know about this issue?

Inter-facility transfer of patients with ST-elevation myocardial infarction (STEMI) are often prolonged due to coordination with emergency medical service (EMS) agencies and percutaneous coronary intervention (PCI) centers and impact patient outcomes.

What was the research question?

What is the contributory role of EMS versus PCI center activation in the timeliness of care for patients transferred with STEMI?

What was the major finding of the study?

Time spent at transferring EDs for patients with STEMI is more dependent on EMS activation than activation of the PCI center.

How does this improve population health?

Encouraging early EMS activation and incorporating this activity into formal policies may improve transfer timeliness for patients with STEMI.

### Data Analysis

We calculated time intervals as the difference between two ED operational timestamps (identified above). When referencing ECGs, we used the ECG diagnostic of STEMI triggering the transfer. As some diagnostic ECGs may be performed by EMS prior to ED arrival, we set the arrival to ECG equal to zero as these visits had access to the diagnostic ECG upon arrival. Since we were using patients with suspected STEMI, and not all patients may have had stent placement, we used the timestamp for initiation of the percutaneous coronary intervention procedure, which was required for inclusion.

We calculated descriptive statistics for patient and facility characteristics, time intervals, and proportion of time intervals of the overall ED length of stay for the overall population, by EMS activation status (before vs after percutaneous coronary intervention activation) and by ED length of stay (≤60 minutes vs >60 minutes). We selected 60 minutes as the cutoff as this was the duration used internally for quality improvement purposes. Group comparisons were conducted using Wilcoxon rank-sum tests for continuous variables and chi-square tests for categorical variables. We used generalized linear models with log link function to quantify relative model ED time intervals of interest (ECG-to-EMS activation) adjusting for patient and facility characteristics, which included patient age, gender, ED mode of arrival (private vs emergency medical services), ED presentation after hours (>5 pm and on weekends), number of facility transfers to VUMC for suspected STEMI in the past year at the time of transfer, and transferring ED facility distance. Analyses were conducted using *R 3.5.1* (R Foundation for Statistical Computing, Vienna, Austria).

## RESULTS

**Figure 1:**
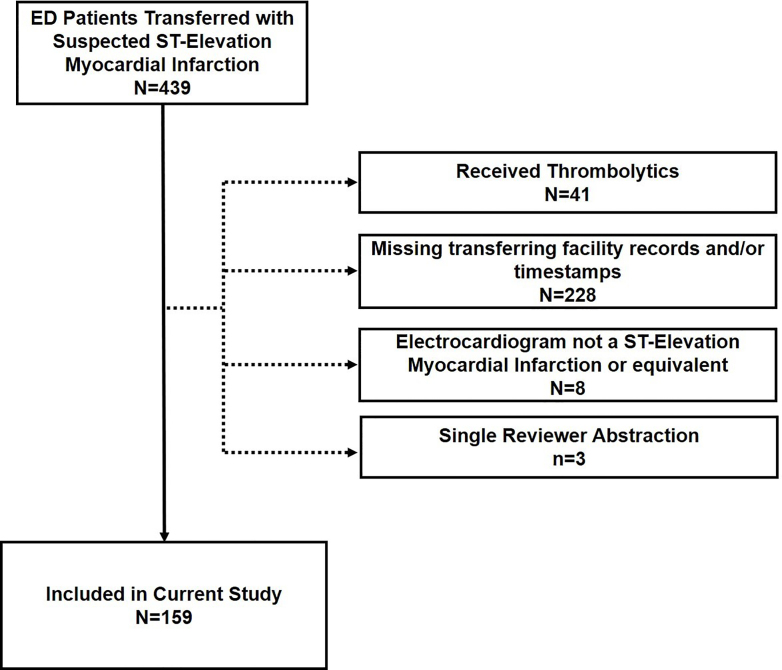
CONSORT Diagram for study population. *ED*, emergency department.

**Figure 2:**
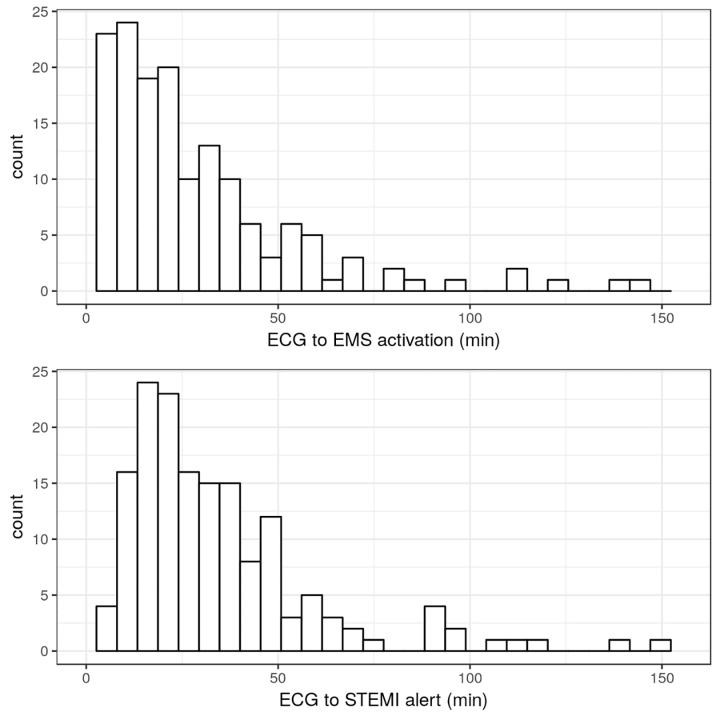
Histogram of electrocardiogram (ECG)-to-emergency medical services activation and ECG-to-percutaneous coronary intervention activation intervals. ECGs used were those diagnostic of ST-elevation myocardial infarction (STEMI). *min*, minutes.

From an initial group of 439 subjects, we identified 159 patients who met inclusion criteria (Figure 1). Subjects were a median of 58 years old (interquartile range [IQR]50, 67), 78% male, and 93% White; 59% arrived to the ED by private vehicle, and 75% presented after hours (Tables 1–2). The median ECG-to-EMS activation interval was 20 minutes (IQR 11, 36), whereas ECG-to-percutaneous coronary intervention activation was 28 minutes (IQR 18, 44) representing 32%, and 46% of the overall ED length of stay, respectively. Transfers with EMS activation first had an 11-minute shorter ECG-to-ED exit interval (61 vs 72 minutes, *P* = 0.047). However, ED arrival-to-percutaneous coronary intervention start was not different when EMS was activated first (108 vs 118 minutes, *P* = 0.07). Among transfers with an ED length of stay ≤60 minutes, 75% (N = 66) had an ECG-to-EMS activation ≤20 minutes. However, only 50% (N = 33) of such transfers with an ED length of stay ≤60 had an ECG-to-percutaneous coronary intervention activation that was ≤20 minutes. Figure 2 shows the relative distribution of these two intervals.

### Multivariable Modeling

Given the relative importance of the ECG-to-EMS activation interval in reducing the ED length of stay, we focused on this interval for the multivariable generalized linear model. Multivariable modeling showed that after-hours presentation was associated with shorter ECG-to-EMS activation (adjusted relative risk [RR] 0.81, 95% confidence interval [CI], 0.76, 0.87, *P*<0.001). Similarly, female gender (adjusted RR 0.82, 95% CI, 0.76, 0.89, *P*<0.001) and increased interfacility transfers in the past year (adjusted RR 0.75, 95% CI 0.71, 0.80, *P*<0.001) were associated with shorter ECG-to-EMS activation (Table 3).

## DISCUSSION

This work advances our understanding of ED interfacility transfer for suspected STEMI patients through two key findings: 1) activating EMS earlier is more likely to reduce the amount of time spent at the transferring ED than percutaneous coronary intervention center activation; and 2) higher transfer volume in the past year, female gender, and after-hours presentations were associated with improved timeliness of EMS activation. These findings support policies that prioritize rapid EMS activation at the transferring ED. Further, these findings suggest that the increased interfacility familiarity that accompanies higher transfer volume may be a modifiable target for intervention to reduce STEMI transfer delays. Through reduction of transfer delays we seek to improve the timeliness of reperfusion as this is essential to optimizing patient outcomes.

Our findings have practical implications for emergency clinicians who must transfer a patient with a suspected STEMI. This research provides evidence to support the clinician’s decision to activate EMS (ie, transportation) prior to calling the percutaneous coronary intervention center. We found that EMS activation can be an important rate-limiting step in the timely transfer of patients with suspected STEMI. Three quarters of all transfers that had an ED length of stay less than 60 minutes had EMS activated in 20 minutes or less. This finding provides additional evidence supporting the use of early EMS activation in clinical practice.^4^

The activation of EMS likely plays such an important role in the transfer process because the patient cannot leave until EMS arrives to physically transport the patient. On the other hand, while percutaneous coronary intervention center activation is important and necessary, the timing of this process appears to be less consequential. Although no formal policy exists at this study setting regarding the activation of EMS, in other settings, some EMS agencies still require an accepting physician name prior to transportation. Auto-acceptance protocols may work by simplifying the transfer process and improving the relationship between organizations by enhancing the likelihood that potential transfers will be accepted by the percutaneous coronary intervention center. As seen in this study, activating EMS prior to the percutaneous coronary intervention center activation was common practice despite no formal policy existing. In settings in which no formal policy exists regarding activating EMS prior to contacting the percutaneous coronary intervention center, incorporating such guidance into transfer center policies and protocols could be a strategy to enhance uptake of early EMS activation.

We also identified that higher transfer volumes in the past year may also have reduced the time to activate EMS. More transfers may indirectly enhance the working relationship between facilities through organizational learning and improved timeliness. Research on interorganizational relationships also suggests that system membership and frequency of transfers may be related with timeliness of care.^11^,^12^ However, with a decreasing incidence of STEMIs,^13^ volume may no longer be sufficient to maintain preparedness and efficiency for transfers. Building higher quality interorganizational relationships may be an alternative strategy in the absence of a sufficient volume of patients who may benefit from timely transfers. Such strategies may include meeting staff/leadership from partner facilities, post-event communication (eg, patient outcome reports), and video communication to enhance interaction.^14^

## LIMITATIONS

Some limitations of this work should be considered. First, we conducted a retrospective analysis of patients with suspected STEMI transferred for primary percutaneous coronary intervention. Not all patients were ultimately diagnosed with STEMI, but the transferring ED and receiving percutaneous coronary intervention center operated as if it were a STEMI. This may, in part, account for our finding of improved timeliness in EMS activation for female patients who typically are less likely to receive percutaneous coronary intervention due to atypical presentations. Further, lack of severity at presentation may confound this finding. To enhance the quality of the retrospective data collection, we used dual abstractor review; however, transferring records might not have been available or potentially conflicting because organizational documentation and charting requirements might have been different (Figure 1). For example, some may have required the collection of specific data elements (eg, physician conversation time) or have a charting template for STEMI. To handle this, we established a hierarchy of quality of evidence. Finally, our study used a single percutaneous coronary intervention center with more than 40 transferring EDs. Evaluation of our findings in other settings is needed to enhance their generalizability and representativeness.

## CONCLUSION

Time spent at transferring EDs for patients with ST-elevation myocardial infarction is more dependent on activating emergency medical services rather than activation of the percutaneous coronary intervention center. Emphasizing this process and formally incorporating it into operational policies may improve transfer timeliness and subsequently reperfusion times.

## Figures and Tables

**Table 1:** Descriptive statistics of percutaneous coronary intervention activation timing relative to emergency medical services activation, and for those transfers above and below 60 minutes by patient demographics.

Variable	EMS Activation After PCI Activation N=28	EMS Activation Before PCI Activation N=131	P-value	ED LOS ≤ 60 min N=66	ED LOS > 60 min N=93	Combined N=159	P-value
Demographics*							
Age, median (IQR)**	55 (48,60)	59 (50,68)	0.11	56 (50,65)	59 (50,67)	58 (50,67)	0.6
Female gender	0.14 (4)	0.24 (31)	0.28	0.21 (14)	0.23 (21)	0.22 (35)	0.84
Race			0.38				0.72
White	0.89 (25)	0.94 (123)		0.94 (62)	0.92 (86)	0.93 (148)	
Black or African American	0.11 (3)	0.06 (8)		0.06 (4)	0.08 (7)	0.07 (11)	
Insurance			0.57				0.37
Private	0.46 (13)	0.36 (47)		0.39 (26)	0.37 (34)	0.38 (60)	
Medicare	0.32 (9)	0.36 (47)		0.29 (19)	0.40 (37)	0.35 (56)	
Medicaid	0.07 (2)	0.05 (6)		0.08 (5)	0.03 (3)	0.05 (8)	
None	0.14 (4)	0.24 (31)		0.24 (16)	0.20 (19)	0.22 (35)	
Private vehicle arrival to ED	0.71 (20)	0.56 (74)	0.14	0.55 (36)	0.62 (58)	0.59 (94)	0.32
Transfers in the past year	9 (3,12)	11 (3,16)	0.25	14.0 (9.0,20.5)	5.0 (2.0,13.0)	10.0 (3.0,16.0)	<0.001
After-hours presentation	0.86 (24)	0.73 (96)	0.17	0.73 (48)	0.77 (72)	0.75 (120)	0.5
Comorbidities							
Hypertension	0.71 (20)	0.68 (89)	0.72	0.70 (46)	0.68 (63)	0.69 (109)	0.79
Smoker	0.43 (12)	0.54 (71)	0.28	0.53 (35)	0.52 (48)	0.52 (83)	0.86
Dyslipidemia	0.68 (19)	0.41 (54)	0.01	0.48 (32)	0.44 (41)	0.46 (73)	0.58
Diabetes	0.39 (11)	0.25 (33)	0.13	0.24 (16)	0.30 (28)	0.28 (44)	0.41
Prior PCI	0.32 (9)	0.16 (21)	0.048	0.14 (9)	0.23 (21)	0.19 (30)	0.15
Prior CABG	0.07 (2)	0.11 (14)	0.57	0.08 (5)	0.12 (11)	0.10 (16)	0.38
Peripheral Artery Disease	0.18 (5)	0.06 (8)	0.039	0.06 (4)	0.10 (9)	0.08 (13)	0.41
Heart Failure	0.04 (1)	0.08 (10)	0.44	0.05 (3)	0.09 (8)	0.07 (11)	0.32
Dialysis	0.00 (0)	0.02 (2)	0.51	0.02 (1)	0.01 (1)	0.01 (2)	0.81
30-Day Mortality	0.04 (1)	0.11 (14)	0.24	0.11 (7)	0.09 (8)	0.09 (15)	0.67

* Demographics are reported in proportion with sample size in parentheses.

** Time intervals are presented in medians with interquartile ranges in parentheses.

*EMS*, emergency medical services; *PCI*, percutaneous coronary intervention; *CABG*, coronary artery bypass graft; *ED*, emergency department; *LOS*, length of stay; *IQR*, interquartile range.

**Table 2:** Descriptive statistics of percutaneous coronary intervention activation timing relative to emergency medical services activation, and for those transfers above and below 60 minutes by time intervals.

Variable	N	EMS activation after PCI activation N=28	EMS activation before PCI activation N=131	P-value	ED LOS ≤ 60 min N=66	ED LOS > 60 min N=93	Combined N=159	P-value
Time intervals								
Total ED LOS	159	68 (59,101)	65 (50,79)	0.083	48.0 (40.0,53.8)	79.0 (68.0,102.0)	65.0 (50.5,84.5)	<0.001
ED arrival to ECG	159	6 (0.25,9.25)	4.00 (0.00,9.00)	0.47	3.00 (−10.75,6.75)	6.00 (2.00,10.00)	5.00 (0.00,9.00)	<0.001
ECG to EMS activation	159	38 (17,61)	19 (9,32)	<0.001	12.0 (7.2,20.0)	31.0 (17.0,51.0)	11.0 (20.0,36.5)	<0.001
ECG to PCI activation	159	32 (17,46)	28 (18,42)	0.85	20 (14,26)	38 (27,53)	28 (18,44)	<0.001
EMS activation to ED exit	159	35 (22,41)	40 (32,49)	0.005	33.5 (28.2,39.0)	45.0 (38.0,52.0)	39.0 (30.5,47.5)	<0.001
PCI activation to ED exit	159	39 (30,46)	30 (22,39)	0.002	26 (18,34)	37 (26,48)	32 (23,39)	<0.001
PCI activation to EMS activation	159	4.5 (0.0,10.2)	−9.0 (−13.0,−5.0)	<0.001	−7.0 (−10.8,−4.0)	−7.0 (−12.0,−2.0)	−7.0 (−12.0,−3.0)	0.86
ECG to ED exit	159	72 (56,91)	61 (46,78)	0.047	46 (40,52)	78 (63,96)	62 (48,82)	<0.001
ED exit to PCI center arrival	159	22 (17,36)	23 (19,32)	0.94	22 (16,27)	25 (20,37)	23 (18,33)	0.007
PCI center arrival to PCI start	158	20 (14,24)	19 (15,24)	0.79	19.0 (16.0,23.8)	19.0 (14.0,24.0)	19.0 (15.0, 24.0)	0.74
ED arrival to PCI start	158	122 (100,158)	112 (93,132)	0.076	89 (77,100)	132 (116,158)	113 (94,140)	<0.001

* Time intervals are presented in medians with interquartile ranges in parentheses.

*EMS*, emergency medical services; *PCI*, percutaneous coronary intervention; *ED*, emergency department; *LOS*, length of stay; *ECG*, electrocardiogram.

**Table 3:** Results from the generalized linear regression models investigating the association of the electrocardiogram-to-emergency medical services activation interval to a priori selected covariates.

Covariate	Univariate			Multivariable		
	
	aOR	95% CI	P	aOR	95% CI	P
Age	0.99	(0.95, 1.03)	0.64	1.02	(0.98, 1.06)	0.39
Transfers to PCI center in past year	0.77	(0.73, 0.81)	<0.001	0.75	(0.71, 0.80)	<0.001
Distance from PCI center	1.17	(1.11, 1.23)	<0.001	1.01	(0.94, 1.07)	0.87
Gender: female vs male (ref)	0.86	(0.80, 0.93)	<0.001	0.82	(0.76, 0.89)	<0.001
Presentation time: after hours vs weekday (ref)	0.82	(0.77, 0.88)	<0.001	0.81	(0.76, 0.87)	<0.001
Mode of transport to ED: EMS vs personal (ref)	0.93	(0.87, 0.99)	0.01	0.94	(0.89, 1.00)	0.06

* Unless otherwise noted, odds ratios for continuous variables are comparing a change from the 25th to the 75th percentile.

*aOR*, adjusted odds ratio; *CI*, confidence interval; *PCI*, percutaneous coronary intervention; *ED*, emergency department; *EMS*, emergency medical services.
